# Neuraxial Anesthesia in Parturients With Intracranial Arteriovenous Malformations: A Case Report

**DOI:** 10.7759/cureus.95223

**Published:** 2025-10-23

**Authors:** Deniz Isikkent, Mohamed Ibrahim, Christy Lee, Rovnat Babazade

**Affiliations:** 1 Department of Anesthesiology, University of Texas Medical Branch at Galveston, Galveston, USA

**Keywords:** cerebral arteriovenous malformation, maternal-fetal medicine, multidisciplinary care approach, neuraxial anesthesia complications, obstetrics and gynecology anesthesia

## Abstract

A cerebral arteriovenous malformation (AVM) is an abnormal nidus of blood vessels that directly connects the arteries and veins in the brain, bypassing the normal capillary network. Due to the rarity of this condition, specific anesthetic management guidelines for patients with intracranial AVMs are limited. In patients with cerebral AVMs, increased intracranial pressure is a concern in the setting of recent hemorrhage, significant mass effect, or venous congestion. In these types of cases, anesthetic management becomes more complex. The risk of AVM rupture during pregnancy, though rare, is a potentially fatal complication and may be considered a relative contraindication to neuraxial anesthesia. We present two cases of pregnant patients with prior diagnosis of AVM who successfully underwent cesarean section using neuraxial anesthesia without any intraoperative or postoperative complications. Neuraxial anesthesia was selected over general anesthesia to minimize hemodynamic fluctuations, reduce intracranial pressure risk, and allow continuous neurological monitoring. These cases add to the limited body of evidence suggesting that neuraxial anesthesia can be a viable option for such patients, provided that comprehensive multidisciplinary care is implemented throughout the preoperative, intraoperative, and postoperative periods.

## Introduction

Cerebral arteriovenous malformations (AVMs) occur when a malformed network of arteries drains directly into the venous system through fistulas, creating an abnormal vascular nidus - a tangle of vessels that bypasses the normal capillary bed [1]. Although AVMs can form in various parts of the body and often remain asymptomatic, cerebral AVMs are of particular concern due to their potential for rupture, which can result in intracranial hemorrhage and subsequent neurological deficits [2]. The etiology of cerebral AVMs is largely congenital, though some cases may develop later in life. The estimated incidence in the general population ranges between 0.001% and 0.5% [3].

During pregnancy, cerebral AVMs account for approximately 8-38% of all cases of intracranial hemorrhage, with reported maternal mortality rates reaching up to 28% and a rebleeding risk of 27-30% in affected patients [4]. Physiological changes of pregnancy - including increased blood volume, cardiac output, and hormonal vascular effects - can exacerbate the hemodynamic stress on fragile intracranial vessels, heightening the risk of rupture. These risks underscore the importance of an extensive multidisciplinary approach involving obstetric, anesthetic, and neurosurgical teams to optimize maternal and fetal outcomes.

Despite the clinical relevance, there remains limited evidence guiding anesthetic management for parturients with cerebral AVMs. The lack of consensus regarding the safest anesthetic technique - neuraxial or general - creates uncertainty in decision-making. Neuraxial anesthesia, which includes spinal, epidural, and combined spinal-epidural (CSE) techniques, is often preferred in obstetrics but poses theoretical concerns in this setting due to potential fluctuations in intracranial pressure and the risk of AVM rupture.

In this case report, we present two term pregnant patients with a prior diagnosis of cerebral AVMs and explore the anesthesia modalities used for each case. We conducted a review of electronic databases to identify pregnant patients with intracranial AVMs who were admitted to our hospital system within the past ten years. Given the rarity of this condition, we identified two relevant cases. Patient consent was obtained prior to accessing the database.

## Case presentation

Case 1

A 27-year-old G3P2 parturient at 39 weeks of gestation was admitted to the Labor and Delivery Unit for a repeat cesarean section. The patient's weight is 69 kg, height is 154 cm, and BMI is 28.7. She had a history of a left occipital AVM measuring approximately 2.4 cm and had previously undergone embolization following a cesarean section at an outside facility. Post-procedure MRI showed a reduction in the size of the AVM. She was subsequently started on anticonvulsant therapy, including levetiracetam and topiramate, to manage seizures and headaches.

**Figure 1:**
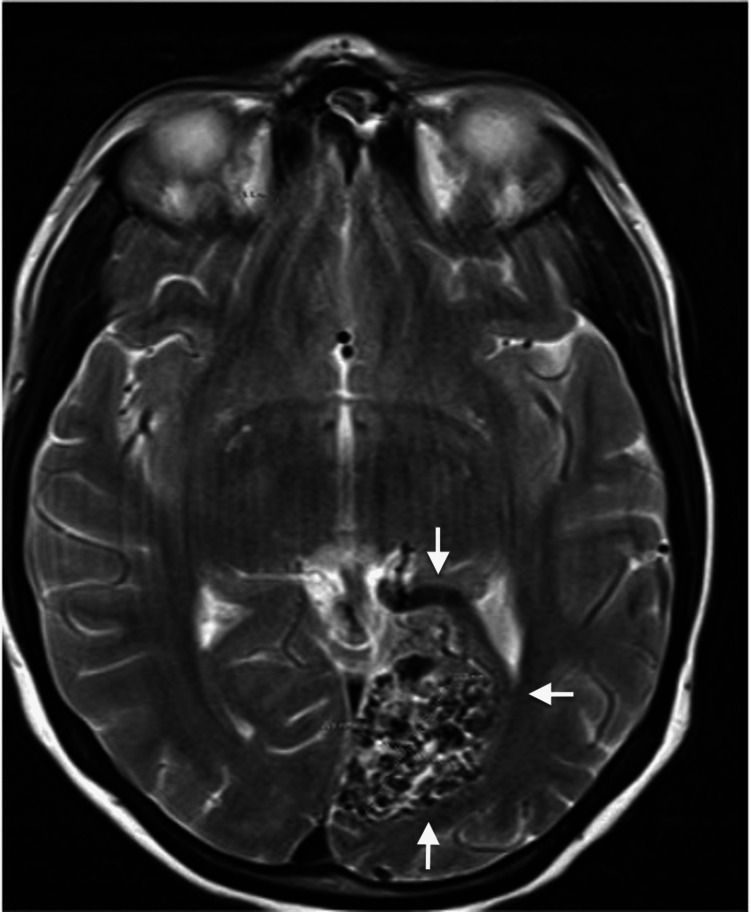
Axial T2-weighted MRI of the brain demonstrating an arteriovenous malformation (AVM) in the medial left occipital lobe involving the calcarine cortex (arrows), measuring approximately 2.9 cm. No evidence of intracranial hemorrhage is present. Minimal perilesional gliosis is noted adjacent to the AVM.

Upon initiating antenatal care, the patient chose to discontinue her anticonvulsant medications, which led to the onset of nausea, vomiting, blurry vision, right-sided weakness, and seizures. A repeat MRI revealed an AVM nidus in the left occipital lobe (Figure 1).

A multidisciplinary plan was developed in collaboration with the maternal-fetal medicine (MFM) and neurology teams. The MFM team's primary goal was to avoid severe hemodynamic changes during the perioperative period. After a thorough preoperative anesthetic evaluation - including assessment of neurological status, seizure history, prior embolization details, and multidisciplinary input from MFM and neurology - our team decided to proceed with neuraxial anesthesia using a combined spinal-epidural (CSE) technique. This approach was chosen to allow for optimal neurological monitoring during the scheduled cesarean section and bilateral tubal ligation while carefully managing the patient’s blood pressure.

In the operating room, the American Society of Anesthesiologists' standard monitors were applied. Antacid and a low, single dose of midazolam were administered preoperatively for anxiolysis, in accordance with standard obstetric anesthesia practice. While the patient was positioned sitting, an uncomplicated CSE technique was performed at the L3-4 level. The intrathecal injection consisted of 12 mg of 0.75% bupivacaine, 150 µg of morphine, and 15 µg of fentanyl. Fentanyl was added to provide rapid-onset intraoperative analgesia, while morphine was included for extended postoperative pain control. The epidural catheter was threaded into the epidural space without complication. A prophylactic phenylephrine infusion was initiated immediately following intrathecal administration to maintain hemodynamic stability, with the goal of keeping mean arterial pressure (MAP) within 20% of baseline. Blood pressure and heart rate were recorded every minute during the first 15 minutes and at five-minute intervals thereafter. No significant hemodynamic fluctuations were observed, and vasopressor support was titrated as needed to maintain the target MAP. The patient remained hemodynamically stable throughout the procedure, and continuous clinical neurological monitoring was performed intraoperatively.

After delivery of the baby, an oxytocin infusion was started at 300 milliunits/min. The newborn had Apgar scores of 7 at one minute and 9 at five minutes. The patient was transferred to the Post-Anesthesia Care Unit (PACU) after the epidural catheter was removed. No notable surgical or anesthetic complications, including postoperative nausea or vomiting, were observed during or after the procedure.

The patient remained hospitalized for 10 days for close neurological observation and multidisciplinary follow-up before being discharged after an uneventful postpartum course. During a follow-up visit two months postpartum, she presented with worsening headaches and seizures, prompting the neurology team to restart her anticonvulsant medications.

Case 2

A G1P0 parturient at 39 weeks of pregnancy was admitted to the Labor and Delivery Unit for a planned cesarean section. The patient's weight is 76 kg, height is 164 cm, and BMI is 28.3. The patient had a history of cerebral AVM, diagnosed during childhood, and underwent gamma knife radiosurgery at ages 5 and 14. Since the procedures, she has had no history of seizures or neurological symptoms, except for occasional mild headaches.

**Figure 2:**
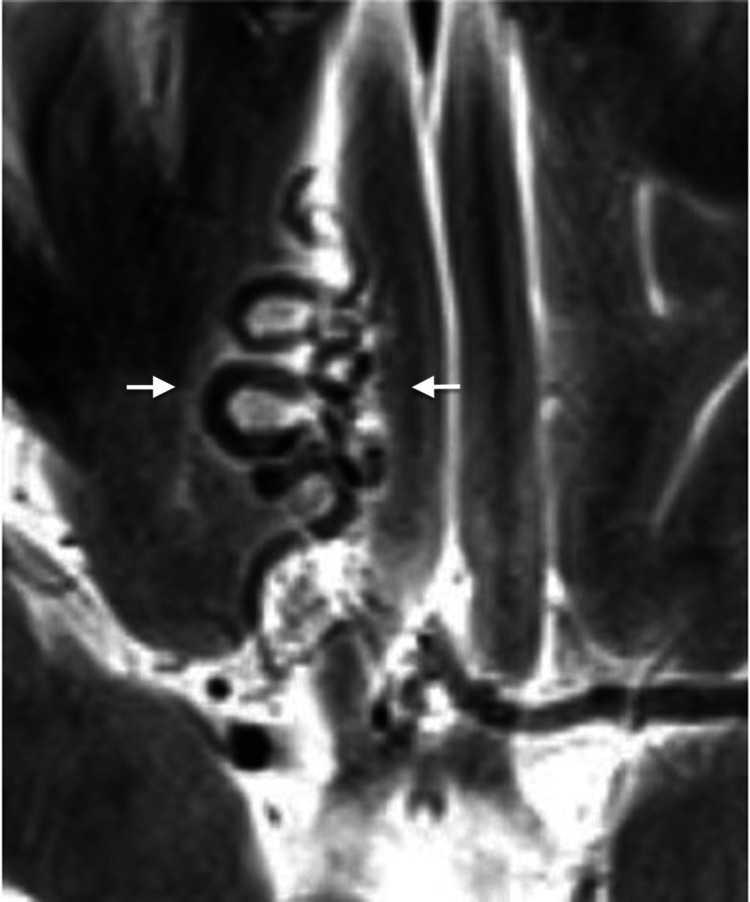
Axial T2-weighted MRI of the brain showing multiple tortuous flow voids in the right gyrus rectus extending toward the right basal ganglia (arrows), consistent with an arteriovenous malformation (AVM) or arteriovenous fistula.

A cerebral MRI (Figure 2) revealed prominent tortuous vessels in the right gyrus region, with possible communication to the right basal ganglia, indicating an abnormality significant for AVM and fistula formation. In preparation for delivery, the anesthesiology team requested a spinal MRI from the cervical to sacral levels, which showed no evidence of AVM formation in the spinal cord.

Multidisciplinary preoperative planning involved obstetric anesthesiology, MFM, neonatology, neurology, and neurosurgery teams. The neurosurgery team was included in the plan in case emergency intervention was needed during surgery.

Following a comprehensive preoperative evaluation - including assessment of neurological status, recent neuroimaging, and seizure history - the multidisciplinary team decided to proceed with epidural anesthesia. Epidural anesthesia was chosen over a single-shot spinal technique to allow for gradual titration of local anesthetic and tighter hemodynamic control, given the patient’s history of intracranial AVM and the need to avoid abrupt fluctuations in cerebrospinal fluid pressure. Preoperatively, the patient was given an oral antacid to reduce gastric acidity and aspiration risk during the peripartum period. In the operating room, standard ASA monitors and an arterial line were applied for continuous, beat-to-beat blood pressure monitoring to ensure tight hemodynamic control and early detection of pressure fluctuations that could predispose to AVM rupture. The epidural catheter was placed in the sitting position at the L3-4 level without complication. A test dose was administered to exclude intravascular or intrathecal placement, followed by fractionated dosing with a total of 15 mL of 2% lidocaine combined with 100 µg of fentanyl and 100 µg of epinephrine. A prophylactic phenylephrine infusion was initiated to maintain MAP within 20% of baseline. The patient’s nausea was promptly relieved with 4 mg of intravenous ondansetron.

After delivery, an oxytocin infusion was initiated at 300 milliunits per minute. The newborn had Apgar scores of 8 and 9 at one and five minutes, respectively. Postoperatively, 3 mg of morphine was administered via the epidural catheter. The catheter was removed without any issues. The patient experienced no complications during the postoperative period and was discharged with instructions for follow-up with neurology, where the plan was to continue conservative management.

## Discussion

Anesthetic management of pregnant patients with a history of arteriovenous malformation remains inconclusive in the literature, largely due to the rarity of the condition. Most clinicians prefer scheduling a cesarean section for patients with AVM, as labor and uterine contractions can potentially increase intracranial pressure, posing a risk to the patient [5]. A retrospective review of pregnant women with untreated, symptomatic intracranial AVMs found that these patients had the same risk of rupture as non-pregnant individuals [6]. Conversely, another study reported an annual hemorrhage rate of 8.1% for cerebral AVMs during pregnancy, which was higher than the rate for non-pregnant patients [7]. These differences likely reflect variations in study populations - particularly whether AVMs were treated or untreated - and the potential influence of gestational timing, as hormonal and hemodynamic changes in late pregnancy and the puerperium may increase rupture susceptibility. These authors recommended that if the AVM was discovered during pregnancy, early intervention should be considered to prevent rupture. However, if the AVM remained intact, comprehensive counseling should be provided, weighing the risks of intervention against the continuation of pregnancy without surgery [8].

In both of our presented cases, the patients had undergone prior neurological interventions, including endovascular embolization and radiosurgery, which reduced the AVM burden but did not achieve complete obliteration. Consequently, they were not considered candidates for additional neurosurgical procedures during pregnancy. This is consistent with previously published case reports describing successful deliveries under epidural, spinal, combined spinal-epidural, and general anesthesia in patients with AVMs, where outcomes were favorable when multidisciplinary planning and tight hemodynamic control were maintained [4].

In our practice, obtaining a full spine MRI is particularly important before performing neuraxial anesthesia in patients with known cerebral AVMs. This imaging ensures there are no associated spinal vascular malformations that could increase the risk of inadvertent puncture or hemorrhage during neuraxial access. Excluding spinal involvement also helps determine the safest level and technique for anesthesia placement, contributing to risk minimization in these complex patients.

In general, obstetric patients are best managed with neuraxial anesthesia if there are no contraindications, as general anesthesia is not typically preferred as a first option. Neuraxial anesthesia is particularly beneficial for pregnant patients with cerebral AVMs, as it avoids the hemodynamic stress associated with laryngoscopy, intubation, and extubation during general anesthesia, all of which could potentially increase intracranial pressure [9]. Positive pressure ventilation associated with general anesthesia can also raise intracranial pressure. Furthermore, neuraxial anesthesia reduces fetal exposure to medications used for the induction and maintenance of general anesthesia and allows for continuous monitoring of the patient's neurological status. A potential risk of epidural anesthesia is an accidental dural puncture, which could increase the risk of AVM bleeding secondary to cerebrospinal fluid leakage and intracranial hypotension [10].

## Conclusions

Due to the limited available literature, we cannot definitively conclude that one method of anesthesia is superior to another for pregnant patients with cerebral AVMs. However, our findings contribute to the growing body of evidence suggesting that neuraxial anesthesia may be considered a viable option in select cases, particularly when supported by appropriate preoperative imaging and multidisciplinary team planning. A coordinated team approach, with effective communication between specialties, can help minimize the risk of morbidity associated with AVM rupture.

In conclusion, while our report is limited to two cases, it highlights that neuraxial anesthesia can be safely performed in carefully selected parturients with cerebral AVMs undergoing cesarean section. Further multicenter or prospective studies are needed to better define the safety and applicability of this approach in antenatal patients with treated AVMs.
